# Comparison between Ringer's lactate and balanced salt solution on postoperative outcomes after phacoemulsification: a systematic review and meta-analysis of randomized controlled trials

**DOI:** 10.31744/einstein_journal/2025RW1569

**Published:** 2025-09-11

**Authors:** Francisco Victor Carvalho Barroso, Dillan Cunha Amaral, Samuel Montenegro Pereira, Ângelo Sergio De Francesco Figueiredo, Júlia Gonçalves Gadelha, Hélio Ferreira Lopes, João Fernando Sobanski, Ricardo Noguera Louzada, David Rocha Lucena

**Affiliations:** 1 Serviço Oftalmológico de Pernambuco Recife PE Brazil Serviço Oftalmológico de Pernambuco, Recife, PE, Brazil.; 2 Universidade Federal do Rio de Janeiro Faculdade de Medicina Rio de Janeiro RJ Brazil Faculdade de Medicina, Universidade Federal do Rio de Janeiro, Rio de Janeiro, RJ, Brazil.; 3 Hospital Geral Dr. Waldemar Alcântara Fortaleza CE Brazil Hospital Geral Dr. Waldemar Alcântara, Fortaleza, CE, Brazil.; 4 Clínica de Olhos De Francesco Fortaleza CE Brazil Clínica de Olhos De Francesco, Fortaleza, CE, Brazil.; 5 Afya Faculdade de Ciências Médicas da Paraíba João Pessoa PB Brazil Afya Faculdade de Ciências Médicas da Paraíba, João Pessoa, PB, Brazil.; 6 Faculdade São Leopoldo Mandic Campinas SP Brazil Faculdade São Leopoldo Mandic, Campinas, SP, Brazil.; 7 Centro Avançado de Oftalmologia Fortaleza CE Brazil Centro Avançado de Oftalmologia, Fortaleza, CE, Brazil.

**Keywords:** Basic saline solution, Saline solution, Ringer's lactate, Phacoemulsification, Cataract extraction

## Abstract

**Introduction:**

The controversy surrounding the potential benefits of the basic saline solution *vs.* Ringer's lactate solution for patients undergoing phacoemulsification for cataracts prompted a systematic review and meta-analysis for comparing the two solutions.

**Objective:**

This systematic review and meta-analysis aimed to compare the basic saline solution with Ringer's lactate for irrigation in phacoemulsification for cataracts.

**Methods:**

We searched the Embase, PubMed, and Cochrane databases for randomized controlled trials comparing basic saline solution with Ringer's lactate in relation to central corneal thickness and loss of endothelial cell density after elective cataract surgery.

**Results:**

Four studies involving 322 patients who underwent phacoemulsification were included in the analysis. Of the participants, 161 (50%) received Ringer's lactate as the irrigation solution, and 161 (50%) received basic saline solution. No differences were noticed between Ringer's lactate and basic saline solution regarding the loss of endothelial cell density within 28 days.

**Conclusion:**

In patients undergoing phacoemulsification for cataracts, no significant differences in the loss of endothelial cell density and changes in central corneal thickness were observed between groups irrigated with Ringer's lactate and balanced salt solution.

**Prospero database registration:** ID CRD42024554821.

## INTRODUCTION

Individuals with age-related eye diseases are increasing in number owing to long life expectancies.^(1)^ According to the World Health Organization (WHO), in 2010, approximately 285 million people worldwide had visual impairment, including 39 million blind people. Approximately 80% of these blind individuals were aged >50 years, and most causes were preventable.^(1)^ During phacoemulsification, certain factors can damage the eyes, such as excessive use of ultrasonic energy, collision of lens nuclear fragments with the corneal endothelium, air bubbles, and an increase in localized temperature.^(2,3)^

During cataract surgery, replacing aqueous fluid with an irrigating solution can affect the survival and function of endothelial cells. The high cost of the basic saline solution (BSS) limits its widespread acceptability and usage.^(4)^ In contrast, although Ringer's lactate (RL) lacks several essential constituents necessary for endothelial functioning and protection, it remains the most widely-used irrigating fluid in low-income countries owing to low cost.^(5,6)^

The corneal endothelium can be efficiently preserved using solutions with a composition similar to that of the aqueous humor.^(7,8)^ After surgery, the corneal thickness increases as the pump and barrier functions of the endothelium are compromised. Therefore, corneal thickness can indicate the extent of surgically-induced endothelial injury.^(9)^ Basic saline solution contains Mg that is essential for the Mg-ATPase endothelial pump, and acetate citrate buffer system, K, Ca, and lactate. These components reduce endothelial cell loss during eye surgery. Several factors contribute to postoperative endothelial cell loss after phacoemulsification, including surgery time, phacoemulsification time, power of ultrasound, instrument-related trauma, size of incision, turbulence of the irrigation solution, and type of intraocular lens and ophthalmic viscosurgical devices (OVDs).^(9)^ With increasing complexity of intraocular surgical techniques, the demand for intraocular irrigation solutions that can preserve the integrity of corneal endothelial cells and other intraocular tissues, even when used in large amounts for long periods, is gradually increasing.^(10)^

Meta-analyses related to the issue mentioned above are scanty, and the latest randomized controlled trial (RCT) does not demonstrate the benefits of BSS over RL.^(2)^

## OBJECTIVE

Therefore, we conducted a systematic review and meta-analysis comparing basic saline solution with Ringer's lactate for irrigation in phacoemulsification for cataracts.

## METHODS

### Eligibility criteria

The key criteria for this meta-analysis were as follows: (1) utilization of RCTs, (2) comparison between RL and BSS, (3) inclusion of patients who have undergone phacoemulsification, (4) incorporation of patients with and without diabetes, and (5) reporting of relevant outcomes. Studies were excluded if they (1) lacked a control group, (2) did not compare changes with respect to the baseline or contained unavailable data, (3) included patients with complicated or extracapsular cataracts, (4) involved patients with corneal disease or previously undergone ocular surgery, or (5) were conducted by inexperienced surgeons.

### Search strategy for identifying studies

We systematically searched the Cochrane Central Register of Controlled Trials, and PubMed and Embase databases from inception to May 2024 using the following terms: ("cataract surgery" OR phacoemulsification) AND ("saline solution" OR bss OR "bss plus") AND ("ringer's solution" OR ringer). Boolean operators (AND, OR) were used to ensure a comprehensive approach.

### Study selection

The data were independently extracted by two authors who also manually searched the references cited in all included studies, and previous systematic reviews and meta-analyses, for any additional studies.

### Data collection and assessment of risk of bias

The quality of the RCTs was assessed using the Cochrane Collaboration tool. Studies were evaluated for risk of bias in five domains: selection, performance, detection, attrition, and reporting biases, and were scored as high, low, or unclear risk. Publication bias was examined through funnel plot analysis of point estimates based on study weights and Egger's regression test.^(11)^

### Data synthesis and analyses

This review and subsequent analysis followed the Cochrane Collaboration and Preferred Reporting Items for Systematic Reviews and Meta-Analyses (PRISMA) guidelines.^(12)^ We focused on changes in central corneal thickness (CCT) in micrometers and loss of endothelial cell density (ECD) in cells/mm^2^. Continuous analyses were used to calculate mean differences, 95% confidence intervals (95%CI), and random effects. Heterogeneity was assessed using the Cochran Q test and I^2^ statistics; p<0.10 and I^2^ >25% were considered significant. Statistical analyses were conducted using Review Manager v.5.4.1. For articles presenting data as medians and interquartile ranges, the data were converted into means and standard deviations using calculators.^(13-15)^

## RESULTS

### Study selection and characteristics

**Figure 1:**
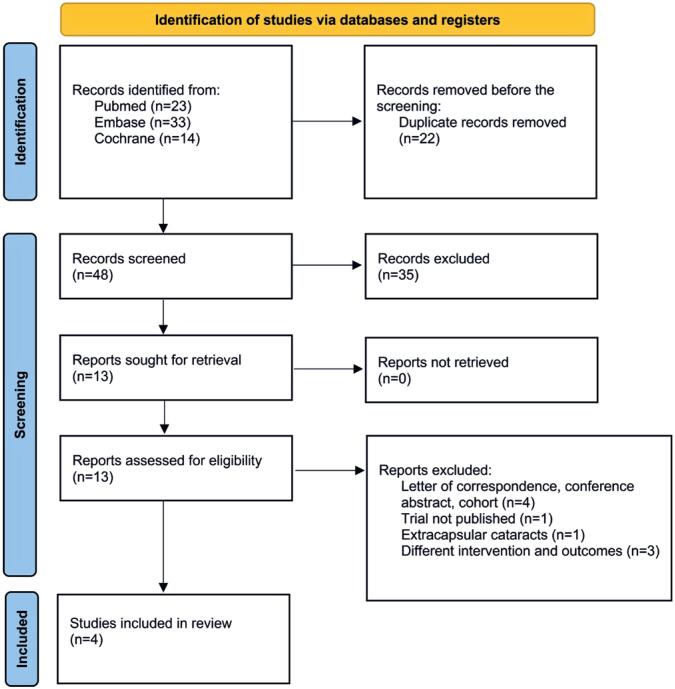
Diagram of study screening and selection

The initial search yielded 70 studies (Figure 1). After removing duplicate records and ineligible studies, 13 were reviewed based on the inclusion criteria. Of these, four were included, totaling 322 eyes of 322 patients. All cases involved age-related uncomplicated cataract surgery with no differences in surgical time. The baseline characteristics of the selected studies (Table 1) indicated that all patients were either diabetic or non-diabetic, with only one exception.^(2)^ Notably, in a study, OVDs were not used during surgery to avoid misinterpretation of endothelial cell protection when comparing between RL and BSS.^(16)^

**Table 1:** Baseline characteristics of the included studies

Studies*	Vasavada et al., 2009^(4)^	Lucena et al., 2011^(17)^	Nayak et al., 2012^(16)^	Rahmawati et al, 2018^(2)^
Population	DM2 +	DM2 +	DM2 +	DM 2 -
Number Ringer/BSS	45/45	55/55	35/35	26/26
Age Ringer/BSS‡	56.5±15.7/58±13.3	65±1.7/63±1.6	N/A	63.19±8.70/61.73±9.11
Female Ringer/BSS	N/A	36/34	N/A	12/13
Cataract density	N/A	Low to medium	Low to medium	N/A
Chop technique	Step-by-step chop *in situ*	Phaco chop	Phaco chop	N/A
Phaco set Up	US, 30-50%; burst, 5-30ms; vacuum, 250-650mmHG; aspiration, 25-30 cc/min	US, 30%; vacuum, 300mmHG, aspiration, 30 cc/min	US maximum, 80%; vacuum, 350-400mmHG; irrigation 60-87cm	N/A
Surgical time (min) Ringer/BSS^†^	5.2±2.76/5.08±2.18	N/A	9.64±2.12/10.27±2.39	6.7±1.71/7.5±1.73
Phaco time (s) Ringer/BSS^†^	53±36/54±23	32.6±4.9/32.9±4.9	53.31±21.21/58.17±20.21	30.11±13.30/29.38±17.65
Phaco machine	Infinity Alcon	Infinity Alcon	Infinity Alcon	N/A
OVD	Viscoat and ProVisc	Methylcelulose	Not utilized	N/A
Pachymeter	Ocuscan Alcon	TopCon	TopCon	TopCon
Specular microscope	Topcon Tokyo	TopCon	TopCon	TopCon

* Randomized clinical trials;

‡ mean±standard deviation.

N/A: not available; BSS: basic saline solution; OVD: ophthalmic viscosurgical device; DM2: type 2 *diabetes mellitus*.

### Long terms outcomes

**Figure 2:**
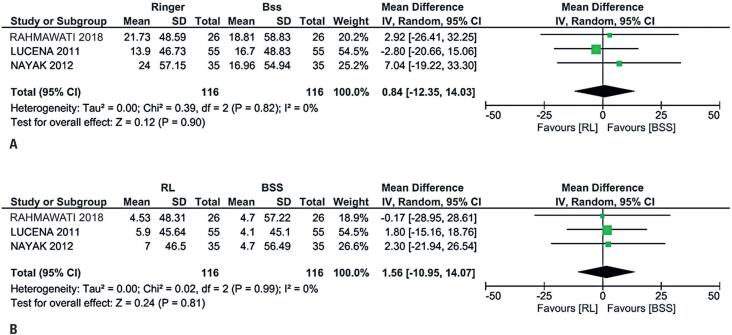
Analyses between Ringer lactate and basic saline solution related to the loss of endothelial cell density. A) No significant difference on the 7^th^ day post-operation; B) No significant difference on the 28^th^ day post-operation

**Figure 3:**
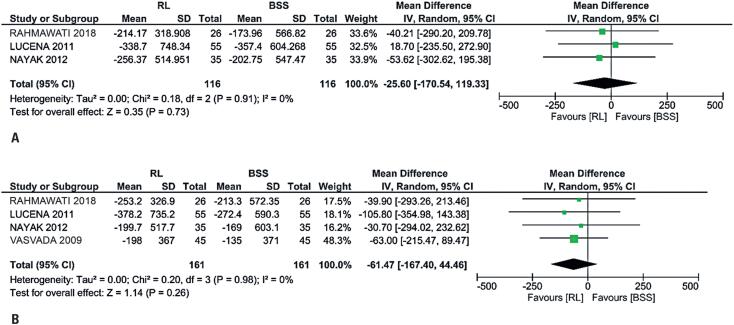
Analyses between Ringer lactate and basic saline solution related to the changes in central corneal thickness. A) No significant difference on the 7^th^ day post-operation day; B) No significant difference on the 28^th^ day post-operation

In the analysis lasting 28 days, no significant differences were observed between RL and BSS in terms of ECDL (mean difference, −61.47 cells/mm^2^; 95%CI= −167.40 to 44.46; p=0.26; I^2^=0%; Figure 3B) and in changes of CCT (mean difference, 1.56μm; 95%CI= −10.95 to 14.07; p=0.81; I^2^=0%; Figure 2B). Three studies provided all data as numerical values for mean difference (MD) and standard deviation (SD).^(2,16,17)^ However, one study did not provide numerical data;^(4)^ therefore, the ECD data were calculated using the formula available in the abstract for determining the percentage loss with low heterogeneity when the data were plotted.

### Short-term outcomes

We were unable to analyze all four RCTs for 7 days postoperatively for ECDL and CCT due to the lack of patient-level data. However, we conducted an analysis for 7 days postoperatively, including only three RCTs, depending on the available data. The results for ECDL showed a mean difference of −25.60 cells/mm^2^ with a 95%CI between −170.54 to 119.33, p=0.73, and I^2^=0% (Figure 3A), and the results for CCT showed a mean difference of 0.84μm with a 95%CI between −12.35 to 14.03, p=0.90, and I^2^ = 0% (Figure 2A).

### Quality assessment

**Figure 4:**
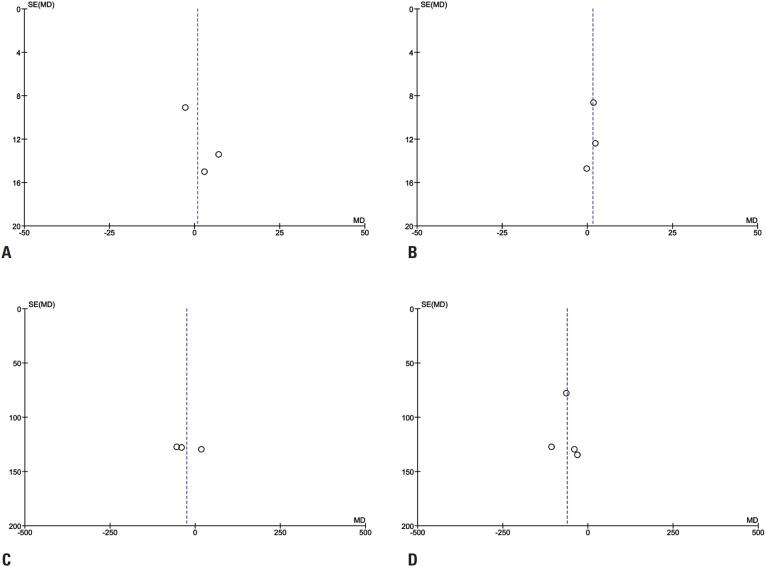
Funnel plot of the analyzed studies. A and B) Funnel plot of endothelial cell density loss analyzes on the 7^th^ and 28^th^ days; C and D) Funnel plot of central corneal thickness analyzes on the 7^th^ and 28^th^ days

**Figure 5:**
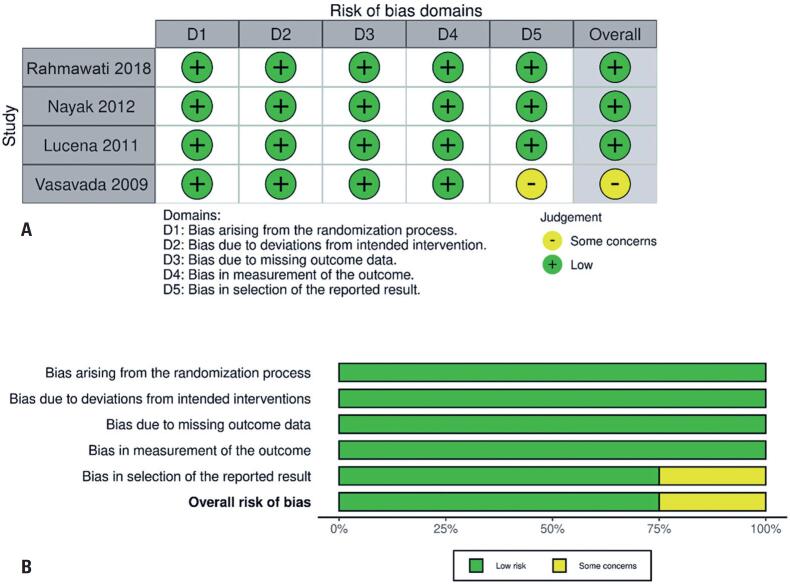
Assessment of publication bias

Individual appraisal of the RCTs is shown in figure 4. The Cochrane Collaboration tool for assessing risk of bias in randomized trials (ROBINS-II)^(18)^ was used to assess the quality of the RCTs. One study did not report the data as numerical values.^(4)^ The other three studies met all criteria for randomization (Figure 4), and no publication bias was noticed. The funnel plot (Figure 5) displayed a symmetrical distribution of similar-weight studies, with convergence towards the pooled prognostic effect size as the weights increased.

## DISCUSSION

We assessed the effectiveness of RL compared with that of BSS irrigation solution during phacoemulsification, based on a comprehensive review and meta-analysis of four studies involving 322 patients. Our long-term findings showed that the use of RL did not significantly differ from that of BSS in terms of the changes in ECD and CCT. Additionally, the short-term analysis did not reveal any benefits of using BSS over RL in CCT and ECD. Although we could not assess short-term results, the functional reserve of a normal endothelium can maintain and reduce long-term corneal thickness despite significant reductions in cell density just days after intraocular surgery.^(9,19)^

Crema et al.^(20)^ have not noticed a significant correlation between ultrasound time and a decrease in endothelial cell percentage; however, other studies have shown conflicting results.^(21,22)^ All RCTs included in our meta-analysis were characterized by the same type of surgical and phaco time, experience of surgeons, and types of cataract density. A relationship between ultrasound energy and endothelial cell loss has been reported.^(21,22)^ Additionally, the type of fracture adopted can affect endothelial cell loss owing to the power of ultrasound. For example, the use of phaco prechop results in a significantly lower mean effective ultrasound time and percentage of postoperative endothelial cell loss than those by the divide-and-conquer technique.^(23)^

All four RCTs were similar in terms of phacoemulsification time, technique, total surgery time, and phaco chop technique. Nayak et al.^(16)^ have not used an OVD; however, the heterogeneity of the results was not compromised. Lucena et al.^(17)^ have reported a trend towards relatively low postoperative endothelial cell density for surgeries with relatively long phacoemulsification times and high irrigation volumes when RL is used.^(17)^ Unfortunately, we could not conduct a sub-analysis of ECD and CT in all four RCTs on 7 postoperative days because of the absence of patient-level data. We were also unable to perform flare analysis and analyze corneal endothelial cells due to differences in analysis methods between studies and subjectivity.

Some studies have reported that BSS and RL are similar in terms of anterior chamber cellularity and flare, as well as in their ability to maintain endothelial cell loss and prevent morphological changes after phacoemulsification in senile cataracts.

## CONCLUSION

Our results do not indicate the superiority of Ringer's lactate to basic saline solution; however, they provide a basis for comparison in meta-analysis for future studies and clinical trials related to these differences in irrigation solutions during phacoemulsification, as well as changes in central corneal thickness and ECDL.
